# Dr. Charles Steele

**Published:** 1914-09

**Authors:** 


### Obituary.

### Dr. CHARLES STEELE.

Charles Steele, M.D., F.R.C.S., who died at the age of 76, on Sept. 20th, at his residence, Richmond Hill, Clifton, was born in 1838, his father having been the Rev. John Steele, Rector of Christ Church, Macclesfield. After an early training in the Isle of Man, he became a student at the Bristol Royal Infirmary and Medical School, and obtained his first qualification, the M.R.C.S., in 1860; the L.R.C.P. followed in 1861. Later, in 1869, he obtained by examination the distinction of F.R.C.S. Eng., and in 1880 the degree of M.D. at Durham. After some junior appointments away from Bristol, he commenced practice in Meridian Place, Clifton, but removed to Richmond Hill a few years later, and practised there for many years, until some six years ago his health gave way, and the infirmities of age compelled him to give up all active occupation.

For forty-five years he did much useful work, and obtained a considerable reputation, so much so that in 1870, on the retirement of Mr. Augustin Prichard, he was elected Surgeon to the Bristol Royal Infirmary. Here he took great interest in his patients, to whom he became much attached, not only by his skill, but also by his geniality and good humour. However, in 1878 he found himself so pressed with work elsewhere, that reluctantly he felt compelled to ask his colleagues to accept his resignation; meanwhile he had shown himself to be a bold and enterprising surgeon, with considerable originality and ingenuity in devising and constructing surgical appliances, amongst which may be mentioned the use of flexible probes, chair supports for spinal curvatures, and steel spring splints. He was at one time on the teaching staff of the Bristol Medical School, when he was appointed to be Lecturer in Physiology. He was also Consulting Surgeon to the Bristol Royal Hospital for Sick Children and Women, and for a short time he acted as Surgeon to the Bristol Police Force. He was for many years a member of our Society, assisting in its meetings.

He was an occasional contributor to the *British Medical Journal* and *Lancet*, where we find records of some of his more remarkable operations, such as excision of the scapula and upper jaw, colotomy, skin grafting, excision of varicose veins, etc.; but these were mostly in the early years of antiseptic surgery, before the modern methods became established.

The present generation knows little of the work of thirty or forty years since: this is soon forgotten, but Charles Steele was in his early years one of the leading surgeons of his time, and he was much beloved by a large clientèle of patients and friends.

The funeral was largely attended by old friends, of whom many had been professional colleagues; amongst these we may mention two of his sons-in-law, Colonel Edward Owen Thurston, I.M.S., and Cyril G. Russ-Wood, F.R.C.S., of Shrewsbury, and his son, Dr. W. Hugh Steele, of Torquay, who is also a distinguished member of the profession, to which Canon Haigh made reference in his remarks at the Church of St. Paul, Clifton, when he said—" Dr. Steele belonged to that church. They valued him. He was one of the devoutest of the devout. He (the speaker) knew what a true man he was . . . They were met to bid him a loving farewell." An old pupil adds "that he was a good conscientious man, honest in his convictions as well as in everything else, and a very hard worker."

